# Perceived Barriers to Home-Based Telemedicine and Facilitators of Public Health Center-Based Telemedicine Among Digitally Capable Community-Dwelling Older Adults: A Cross-Sectional Scenario-Based Survey

**DOI:** 10.3390/healthcare14162660

**Published:** 2026-08-21

**Authors:** Seung-Kwon Ji, Wonseuk Jang

**Affiliations:** Department of Medical Device Engineering and Management, Yonsei University College of Medicine, 50-1 Yonsei-ro, Seodaemun-gu, Seoul 03722, Republic of Korea; cooltouch7@yonsei.ac.kr

**Keywords:** telemedicine, older adults, public health center, digital literacy, intention to use, assisted telemedicine, scenario-based survey

## Abstract

**Background/Objectives:** Home-based telemedicine requires patients to perform technical tasks independently, whereas assisted public health center (PHC)-based telemedicine transfers some of these tasks to on-site personnel. This study describes model-specific perceptions and estimates the difference in intention to use between the two scenarios among relatively digitally capable, community-dwelling older adults. **Methods:** A cross-sectional, fixed-order, scenario-based online survey was completed by 300 adults aged 65 years or older in South Korea. Home-based barriers and PHC-based facilitators were analyzed as non-equivalent descriptive domains. The primary paired comparison used three conceptually matched intention items. Internal consistency, exploratory factor evidence, 95% confidence intervals (CIs), Pearson and Spearman correlations, and multivariable linear regressions with HC3 robust standard errors were reported. **Results:** The seven-item home-based barrier score was 2.80 (SD 0.76; 95% CI 2.71–2.89), and the seven-item PHC facilitator score was 3.58 (SD 0.58; 95% CI 3.52–3.65). Intention scores were 3.91 (SD 0.64) for home-based and 3.88 (SD 0.66) for PHC-based telemedicine. The paired mean difference (PHC minus home) was −0.030 (95% CI −0.107 to 0.047; *p* = 0.441; Cohen’s dz = −0.045, bootstrap 95% CI −0.155 to 0.071). Preferences were home-based 42.7% (95% CI 37.2–48.3%), PHC-based 41.3% (95% CI 35.9–47.0%), and unsure 16.0% (95% CI 12.3–20.6%). After adjustment, digital literacy remained positively associated with both intention outcomes. **Conclusions:** No statistically detectable difference in intention to use was observed between the two hypothetical scenarios; this finding does not establish equivalence. The item-level results generate a hypothesis that staff-supported PHC delivery may be useful for some older adults, but actual uptake, effectiveness, accessibility, and equity require evaluation in real-world studies that include less digitally capable and more vulnerable populations.

## 1. Introduction

Population aging has become a major challenge for healthcare systems worldwide. As the number of older adults increases, healthcare systems face growing demand for continuous management of chronic diseases, repeated medical consultations, and long-term monitoring. Many older adults live with conditions such as hypertension, diabetes, cardiovascular disease, musculoskeletal disorders, or multimorbidity, all of which require sustained contact with healthcare providers rather than one-time treatment. At the same time, physical frailty, limited mobility, geographic isolation, and transportation difficulties may restrict access to conventional face-to-face care. These challenges highlight the need for care delivery models that can support continuous, accessible, and equitable healthcare for older adults.

Telemedicine can enable consultation, monitoring, and follow-up without requiring patients to visit healthcare facilities as frequently [1]. Its use expanded rapidly during the COVID-19 pandemic, but availability did not translate into equal access or use [1,2]. Older adults, particularly those with disability, limited technology experience, lower education, or other social disadvantages, may remain unready for or less likely to use video-based telemedicine [3,4]. The practical question is therefore not only whether telemedicine is available, but whether its delivery model matches users’ capabilities, preferences, and support needs.

A common form of telemedicine is the home-based model, in which patients independently use smartphones, computers, or medical devices to measure health information, transmit data, and communicate with healthcare providers. This model may be convenient for digitally capable users, but it assumes that patients can operate devices, manage internet connections, and respond to technical problems. Sensory, cognitive, communication, and technology-related limitations can impede independent use among older adults [3,4,5]. Concerns about immediate assistance, measurement accuracy, privacy, and remote communication may also affect anticipated use.

One possible service configuration is assisted telemedicine delivered through a community health facility. This study focuses on a public health center (PHC)-based scenario in which personnel assist with device operation, health measurement, data transmission, and connection to a remote clinician. Korean community health centers have been used for chronic disease services involving older adults [6]. In the present study, however, the PHC model was hypothetical; no actual service, accessibility, or clinical effectiveness was evaluated.

**Figure 1:**
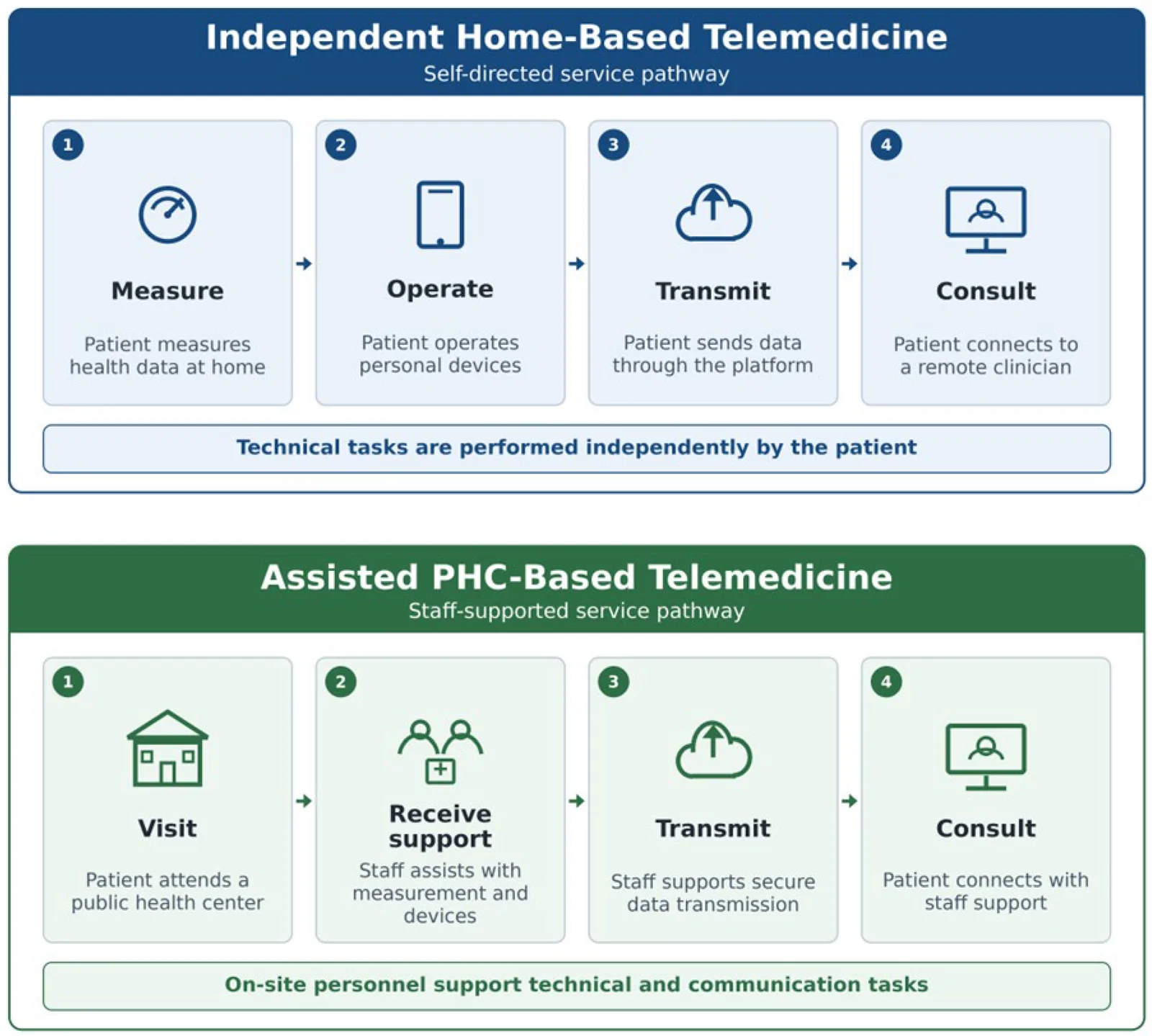
Service-pathway comparison of independent home-based and assisted public health center (PHC)-based telemedicine.

The service workflows of independent home-based and assisted PHC-based telemedicine are summarized in Figure 1.

The two scenarios differed in where care occurred and who performed the technical tasks. The figure describes workflow only and does not imply that either model is superior.

Assisted telemedicine is consistent with a sociotechnical view in which outcomes depend on interactions among the patient, staff, technology, healthcare organization, and wider system [7]. Community facilities may therefore be considered as one possible implementation setting, but feasibility depends on staffing, privacy, workflow, financing, and local accessibility.

Previous research has examined telehealth use in geriatric care, barriers and disparities, telecare among community-dwelling older adults, and the complexity of remote service implementation [1,2,3,4,5,7]. Less is known about how older adults respond to a hypothetical assisted PHC pathway alongside independent home-based telemedicine. Because the present questionnaire used differently framed model-specific items, the study describes home-based barriers and PHC-based facilitators separately and restricts direct comparison to matched intention items.

This study aimed to (1) describe perceived barriers to independent home-based telemedicine; (2) describe perceived facilitators of an assisted PHC-based scenario; (3) estimate the paired difference in intention to use using three conceptually matched items; (4) describe model preference; and (5) examine associations of digital device literacy with the principal outcomes before and after adjustment. The results are limited to relatively digitally capable, community-dwelling older adults who could complete an online survey independently.

## 2. Theoretical Background and Hypotheses

### 2.1. Innovation Resistance in Telemedicine for Older Adults

Innovation Resistance Theory proposes that resistance can arise from perceived risk, incompatibility with established routines, and uncertainty, rather than simply representing the absence of acceptance [8]. For independent telemedicine, unfamiliar device operation, data transmission, remote communication, and lack of immediate assistance may create practical or psychological resistance.

For older adults, resistance to telemedicine may be shaped by several barriers. Functional barriers may arise when the service is perceived as difficult to use or inconsistent with existing healthcare habits. Risk barriers may involve concerns about inaccurate measurement, privacy and data security, or the possibility that symptoms may not be properly communicated to physicians. Psychological barriers may also emerge from anxiety about using technology, low confidence in digital skills, or a preference for direct in-person interaction. These barriers are particularly relevant in home-based telemedicine, where older adults are expected to use digital devices independently and manage the care process without direct professional support.

However, the extent of perceived barriers may vary depending on the characteristics of the user population. Older adults who are relatively familiar with smartphones, mobile applications, internet services, or self-measurement devices may not necessarily report strong overall resistance to home-based telemedicine. Nevertheless, even digitally capable older adults may still experience specific concerns, such as a lack of immediate assistance, uncertainty about measurement accuracy, privacy protection, and difficulty communicating health status remotely. Therefore, this study examines perceived barriers to independent home-based telemedicine as a model-specific perception domain.

**Hypothesis** **1 (H1).** *Older adults will report identifiable perceived barriers to independent home-based telemedicine, particularly in relation to immediate assistance, measurement reliability, privacy protection, and communication with physicians.*

### 2.2. Assisted Telemedicine and the Role of Human Intermediaries

The concept of mediated or assisted telemedicine suggests that technology acceptance is influenced not only by an individual’s technical ability, but also by the availability of support within the service environment. In a public health center (PHC)-based telemedicine model, healthcare personnel can assist older adults with device operation, measurement of health information, data transmission, and connection to remote physicians. In this setting, part of the technical burden is transferred from the patient to trained personnel.

The availability of personnel and technical support corresponds to facilitating conditions in UTAUT [9], while perceived ease of use and usefulness are central to TAM [10]. In this study, these theories informed the selection of model-specific descriptive domains; the questionnaire was not designed as a complete operationalization or formal test of either theory.

In the PHC-based model, human intermediaries may function as “digital health facilitators” by reducing technical uncertainty, supporting communication with physicians, increasing trust in measurement accuracy, and providing immediate assistance when problems occur. Therefore, rather than measuring the PHC-based model as the direct opposite of resistance, this study conceptualizes it as an assisted service model that may generate perceived facilitators and acceptability among older adults.

**Hypothesis** **2 (H2).** *Older adults will report positive perceived facilitators and acceptability for PHC-based telemedicine, particularly in relation to professional assistance, communication support, perceived safety, and measurement reliability.*

### 2.3. Intention to Use Home-Based and PHC-Based Telemedicine

In technology acceptance research, intention to use is a key indicator of likely adoption. TAM links perceived ease of use and perceived usefulness with behavioral intention [10]. In this study, the home-based and PHC-based scenarios may elicit intention through different anticipated service features; actual adoption was not observed.

Home-based telemedicine may be attractive to older adults who value convenience, independence, reduced travel, and the ability to receive care from home. This model may be especially acceptable to older adults who are digitally capable and confident in using devices. In contrast, PHC-based telemedicine may be attractive to older adults who value professional assistance, communication support, measurement reliability, and immediate help when problems occur. Therefore, the two models should not necessarily be understood as competing alternatives, but as different service pathways that may meet different user needs.

Accordingly, this study compares older adults’ intentions to use independent home-based telemedicine and assisted PHC-based telemedicine. Rather than assuming that one model will be universally preferred, this study examines whether intention to use differs between the two models and whether both models may be acceptable to older adults.

**Hypothesis** **3 (H3).** *Intention to use will differ between home-based and PHC-based telemedicine models.*

### 2.4. Digital Device Literacy and Telemedicine Acceptance

Digital device literacy may influence comfort with smartphones, applications, internet services, and self-measurement devices. Prior Korean research has linked perceived quality characteristics with older adults’ acceptance intentions for healthcare kiosks [11]. In independent home-based telemedicine, digital capability may be particularly relevant because the patient performs the technical tasks without on-site assistance.

For the assisted PHC scenario, staff support could theoretically reduce some patient-performed technical tasks. Nevertheless, this study did not experimentally test whether assistance attenuates the effect of digital literacy, and differences between correlation coefficients were not formally compared. Associations for each scenario were therefore estimated separately and interpreted without claiming that one association was stronger than another.

**Hypothesis** **4 (H4).** *Digital device literacy will be associated with home-based barriers and with intention to use each scenario. Associations with the PHC facilitator domain will be examined exploratorily.*

## 3. Materials and Methods

### 3.1. Study Design

This study employed a cross-sectional, scenario-based survey design to examine older adults’ perceptions of two telemedicine delivery models: independent home-based telemedicine and assisted public health center (PHC)-based telemedicine. A within-subject design was adopted, in which each participant evaluated both scenarios. This design allowed for the comparison of perceived intention to use between the two models while reducing inter-individual variability related to demographic characteristics, prior technology experience, and personal attitudes toward healthcare services.

The two scenarios were presented using standardized written descriptions. Scenario A described a home-based telemedicine model in which older adults independently use digital devices and communicate remotely with physicians from their homes. Scenario B described a PHC-based telemedicine model in which older adults visit a nearby public health center and receive assistance from healthcare personnel during health measurement, device operation, and connection to a remote physician.

The home-based scenario was followed by the PHC-based scenario in a fixed order; neither scenario order nor item-block order was randomized or counterbalanced. The home items were predominantly negatively framed barriers, whereas the PHC items were positively framed facilitators. Consequently, the two domain scores are non-equivalent and were not compared numerically. The survey-programming document recorded a 10 s control immediately before the first scenario-related response block; the materials available to the authors did not document further platform-level implementation details. Fixed order, contrast, acquiescence, and framing effects are treated as major design limitations.

### 3.2. Participants and Data Collection

The study population consisted of community-dwelling adults aged 65 years or older residing in South Korea. Data were collected from 1 July to 8 July 2026 through an online survey administered by a professional survey agency after IRB approval. No quota sampling was used. Eligibility required age 65 years or older, community residence, ability to read and understand the online study information, ability to complete the questionnaire independently, and voluntary electronic consent. The survey program terminated respondents who did not meet these conditions before the main questionnaire. The survey introduction stated an expected completion time of approximately 30–40 min; actual response durations were not included in the delivered dataset. These criteria necessarily excluded individuals unable to complete the survey without assistance.

The survey-programming document specified a recruitment target of at least 100 valid responses and a maximum of 300. Recruitment continued until the prespecified upper limit of 300 valid responses was reached, and all 300 were analyzed. No formal a priori effect-size calculation was specified. As an achieved-sample sensitivity analysis, N = 300 provided 80% power at a two-sided alpha of 0.05 to detect a paired standardized mean difference of approximately |dz| = 0.162; this analysis is not presented as an a priori justification.

**Figure 2:**
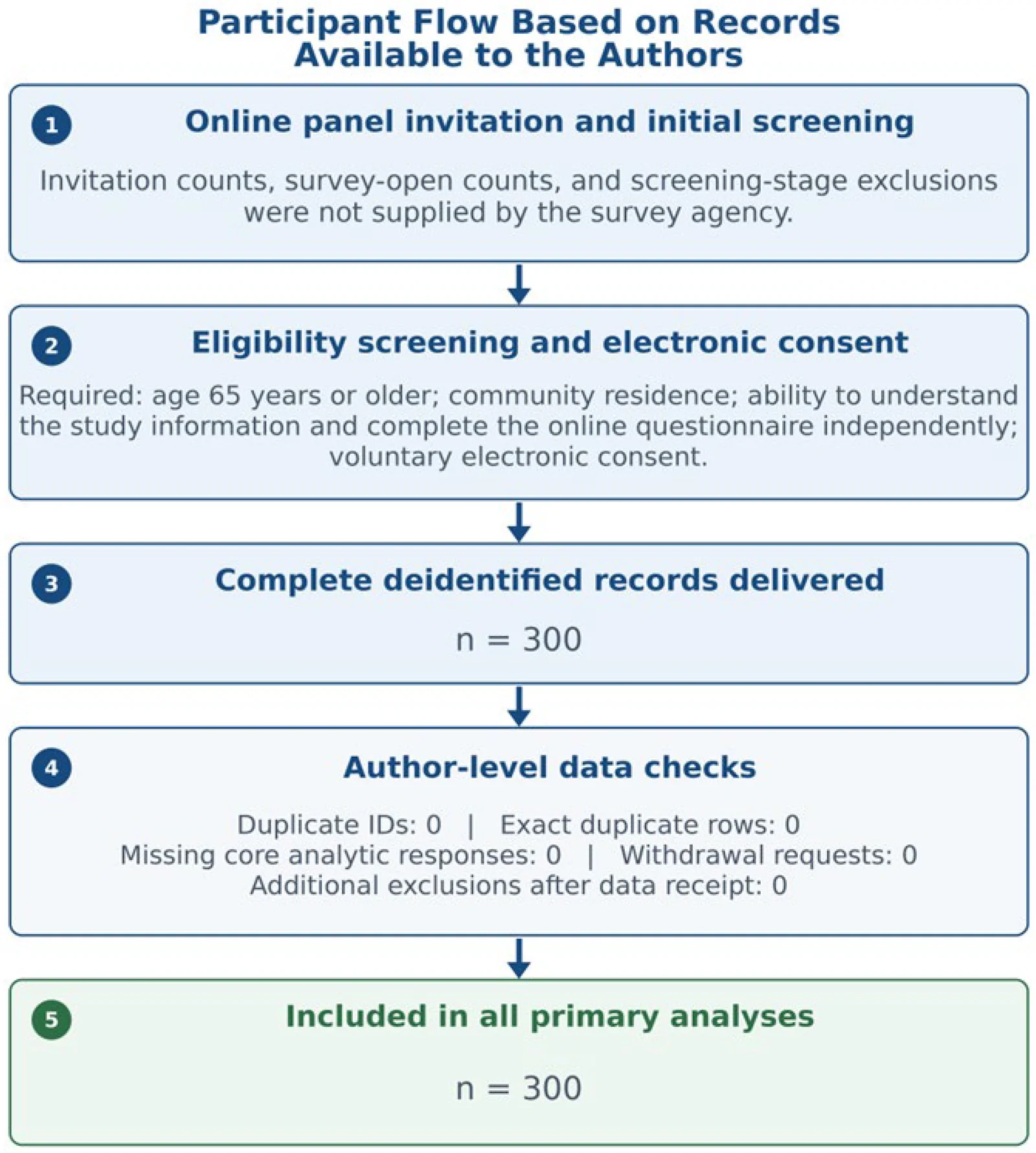
Participant flow based on records available to the authors.

The agency delivered a deidentified dataset containing 300 complete records that had passed programmed eligibility and consent screening. Counts of panel members invited, unique survey visitors, individuals opening or starting the survey, and screening-stage exclusions were not supplied. Accordingly, the CHERRIES view, participation, and completion rates could not be calculated, and sequential case identifiers were not used as denominators. The delivered dataset contained no duplicate IDs, exact duplicate rows, missing values in core analytic variables, or withdrawal requests, and the authors excluded no additional records. Actual response-duration data, the technical method used to prevent multiple submissions, agency-level speeding or straight-lining thresholds, the complete quality-control log, and incentive details were not available in the survey materials provided to the authors. The available participant flow is shown in Figure 2.

The questionnaire collected information on demographic characteristics, prior awareness and experience of telemedicine, perceived barriers to home-based telemedicine, perceived facilitators and acceptability of PHC-based telemedicine, intention to use each model, digital device literacy, model preference, reasons for preferring the PHC-based model, and open-ended concerns regarding telemedicine use.

### 3.3. Scenario Definitions

Participants were presented with two hypothetical telemedicine scenarios.

Scenario A, Home-Based Telemedicine, described a model in which an older adult independently uses digital devices, such as a smartphone, computer, blood pressure monitor, or blood glucose meter, at home to measure health information and transmit the results to a physician. The consultation occurs through video or audio communication, and no direct in-person assistance from healthcare personnel is provided during the process.

Scenario B, PHC-Based Telemedicine, described a model in which an older adult visits a nearby public health center. A nurse or other healthcare personnel assists with device operation, health data measurement, and connection to a remote physician. In this scenario, the participant receives in-person support during the telemedicine process.

These two scenarios were designed to contrast an independent use model with an assisted use model. The home-based model emphasizes individual operation and self-management, whereas the PHC-based model emphasizes professional assistance, institutional support, and mediated access to remote care.

### 3.4. Measures and Instrumentation

The study-specific questionnaire was informed by Innovation Resistance Theory, TAM, UTAUT, and literature on telemedicine and digital health use. It was not a previously validated instrument. No documented expert content-validity rating, cognitive interview, pilot test, translation validation, or test–retest assessment was available. All perception items used a 5-point Likert scale from 1 (strongly disagree) to 5 (strongly agree). Item-level estimates are therefore emphasized, and the factor analyses reported below are exploratory evidence from the same analytic sample rather than independent validation.

The full questionnaire, including the original Korean survey items with English translation notes, is provided as Supplementary File S1.

Perceived barriers to home-based telemedicine were summarized using seven items (A1-A7: burden, device difficulty, measurement concern, communication difficulty, privacy concern, lack of immediate help, and psychological discomfort). Item A8 (“Overall, I would not want to use home-based telemedicine”) was reported descriptively but excluded from the barrier composite because it reflects negative behavioral intention rather than a specific barrier. Higher seven-item scores indicated greater perceived barriers.

Seven PHC items (B1–B7) were reported primarily as separate descriptive indicators covering lower burden, assistance with device use, trust in measurement, communication support, privacy, immediate help, and psychological acceptance. A seven-item mean was retained as an exploratory facilitator composite only after examining its internal consistency and one-factor structure; it was not treated as the inverse of the home-based barrier score.

Intention to use was assessed separately for each scenario. The primary score for each model used three matched items: willingness to use if needed, willingness to use if cost was appropriate, and perceived usefulness for chronic disease management. The home item concerning social recommendation and the PHC item concerning proximity were reported as questionnaire items but excluded from all direct comparisons because they represented different contextual conditions.

The three-item intention scores were the only outcomes compared directly between scenarios. The four-item comparison was removed because the recommendation and proximity items were not conceptually interchangeable.

Digital device literacy was assessed using six items covering familiarity with smartphone use, ability to install and use mobile applications, comfort with text messaging or mobile messengers, ability to use measurement devices such as blood pressure or blood glucose monitors, ability to access and search the internet, and overall confidence in using digital devices. Higher scores indicated higher digital device literacy.

Participants were also asked to indicate their preferred telemedicine model: home-based telemedicine, PHC-based telemedicine, or not sure. In addition, participants were asked to select reasons for preferring PHC-based telemedicine. Because this item was answered by all participants, the analysis of reasons for preferring PHC-based telemedicine was restricted to participants who selected PHC-based telemedicine as their preferred model. An open-ended item was included to allow participants to describe their major concerns regarding telemedicine use.

### 3.5. Statistical Analysis

Initial data management and descriptive analyses were performed using IBM SPSS Statistics 27.0 (IBM Corp., Armonk, NY, USA). Additional confidence intervals, factor analyses, sensitivity analyses, and HC3 regression estimates were produced in Python 3.12 using SciPy 1.17.0 and scikit-learn 1.8.0. Two-sided *p* values below 0.05 were considered statistically significant.

Internal consistency was estimated using Cronbach’s alpha and one-factor McDonald’s omega. Exploratory dimensionality was evaluated using the Kaiser–Meyer–Olkin (KMO) statistic, Bartlett’s test of sphericity, maximum-likelihood exploratory factor analysis of standardized items, and parallel analysis with 1000 random datasets. Because these analyses used the same sample in which outcomes were estimated, they provide preliminary structural evidence and do not establish content, criterion, or external construct validity.

Home-based barriers, PHC-based facilitators, and each individual item were summarized with means, standard deviations, and 95% CIs. The two domain composites were never compared because their framing and content were not symmetrical.

The paired comparison used only the matched three-item intention scores. The PHC-minus-home mean difference was reported with a 95% CI, together with the paired t statistic and Cohen’s dz. The 95% CI for dz was obtained from 20,000 bootstrap resamples. No equivalence margin was prespecified and no equivalence test was performed; a non-significant result was interpreted only as no statistically detectable difference.

Pearson correlations with Fisher-transformed 95% CIs and Spearman rank correlations were calculated between digital literacy and the four principal outcomes. Two multivariable linear regression models estimated home-based and PHC-based intention separately. Each model included its model-specific perception score, digital literacy, age, sex, education (college or higher), any chronic disease, telemedicine awareness, prior telemedicine use, residential region, and living arrangement. HC3 robust standard errors and 95% CIs were used. Variance inflation factors, Breusch–Pagan tests, and residual Shapiro–Wilk tests were examined. A separate healthcare-device-experience variable was not collected; ability to use measurement devices was one component of the digital literacy score and was not entered simultaneously as an additional covariate.

Model preference and reasons for PHC preference were summarized with Wilson 95% CIs for proportions. Reasons were restricted to participants selecting PHC-based telemedicine. All core analytic variables were complete, so no imputation was performed. The study is reported in accordance with the STROBE statement [12], and the completed checklist is provided as Supplementary File S2. A CHERRIES-aligned inventory of available and unavailable Internet-survey reporting information is provided as Supplementary File S4.

## 4. Results

Participant characteristics are presented in Table 1. All 300 delivered records satisfied the analytic inclusion criteria and were included in every primary analysis.

### 4.1. Participant Characteristics

The sample included 300 community-dwelling older adults aged 65–85 years (mean 68.93, SD 3.48; 95% CI 68.53–69.32). Participants were predominantly aged 65–69 years (62.7%); only five (1.7%) were aged 80 years or older. There were 179 men (59.7%) and 121 women (40.3%). Residential distribution was Seoul 24.3%, Gyeonggi 31.0%, metropolitan cities 18.7%, and other provinces 26.0%; rurality itself was not measured.

The sample was highly selected: 73.0% had college-level education or higher, 92.0% had previously heard of telemedicine, and the mean digital literacy score was 4.11/5. These characteristics should be considered when interpreting all estimates.

Chronic health conditions were common among the participants. Overall, 223 participants (74.3%) reported having at least one chronic disease. Hypertension was the most frequently reported condition (42.3%), followed by cardiovascular disease (19.0%), diabetes (18.3%), musculoskeletal disease (14.0%), other chronic diseases (16.0%), and respiratory disease (4.0%). These findings indicate that the study sample included older adults with a practical need for repeated monitoring and ongoing healthcare contact.

Most participants had heard of telemedicine (92.0%), but only 5.0% had used it. Mean digital literacy was 4.11 (SD 0.62; 95% CI 4.04–4.18), and only 18 participants (6.0%) scored 3.0 or lower. The results therefore characterize relatively digitally capable older adults able to complete an online survey independently, not older adults broadly or those most affected by digital exclusion.

### 4.2. Perceived Barriers to Home-Based Telemedicine

The seven-item home-based barrier scale had alpha = 0.882 and omega = 0.884. KMO was 0.887, Bartlett’s chi-square(21) = 1016.35 (*p* < 0.001), parallel analysis retained one factor, and loadings ranged from 0.547 to 0.811. The mean seven-item score was 2.80 (SD 0.76; 95% CI 2.71–2.89). A8 was not included in this composite. Item-level results are presented in Table 2.

The highest item was difficulty receiving immediate help (A6: 3.38, 95% CI 3.26–3.50), followed by concern about measurement accuracy (A3: 3.07, 95% CI 2.96–3.18) and privacy (A5: 3.03, 95% CI 2.92–3.15). Device-operation difficulty was lower (A2: 2.32, 95% CI 2.21–2.43). These are item-level perceptions within a hypothetical scenario and should not be interpreted as observed barriers during actual service use.

These findings partially support Hypothesis 1. Rather than indicating strong overall resistance to home-based telemedicine, the results suggest that older adults perceived specific barriers related to immediate assistance, measurement reliability, privacy protection, and communication during independent home-based use.

### 4.3. PHC-Based Telemedicine Facilitator Ratings

The seven PHC facilitator items had alpha = 0.863 and omega = 0.864. KMO was 0.867, Bartlett’s chi-square(21) = 859.80 (*p* < 0.001), parallel analysis retained one factor, and loadings ranged from 0.532 to 0.794. The exploratory composite mean was 3.58 (SD 0.58; 95% CI 3.52–3.65). This score was not compared with the home barrier score. Item-level results are presented in Table 3.

The highest-rated PHC item was assistance with device use (B2: 3.95, 95% CI 3.87–4.03), followed by communication support (B4: 3.81, 95% CI 3.72–3.89) and immediate help/safety (B6: 3.67, 95% CI 3.58–3.76). These responses indicate positive anticipated perceptions of the described scenario, not demonstrated implementation benefits.

The item-level responses were directionally consistent with Hypothesis 2, but the fixed positive framing of the PHC items and the hypothetical design preclude a stronger inference about comparative acceptability or actual support benefits.

### 4.4. Comparison of Intention to Use Between Models

The matched three-item intention scales showed alpha = 0.851 and omega = 0.854 for home-based telemedicine and alpha = 0.867 and omega = 0.868 for PHC-based telemedicine. Parallel analysis retained one factor for each scale; loadings were 0.767–0.874 and 0.811–0.858, respectively.

Only the three matched intention items were used in the primary paired analysis. The non-equivalent recommendation and proximity items were excluded, and the previous four-item sensitivity comparison was removed. The paired comparison is presented in Table 4.

The home-based intention mean was 3.911 (SD 0.637; 95% CI 3.839–3.984) and the PHC-based mean was 3.881 (SD 0.658; 95% CI 3.806–3.956). The paired difference was −0.030 (95% CI −0.107 to 0.047), t(299) = −0.771, *p* = 0.441. Cohen’s dz was −0.045 (bootstrap 95% CI −0.155 to 0.071). Thus, the analysis did not detect a statistically significant difference.

Because no equivalence margin was defined, the confidence interval and non-significant *p* value do not establish equivalence, interchangeability, or similar acceptability. They indicate only that this sample and analysis did not detect a difference in matched intention scores.

Intention was relatively high under both hypothetical descriptions, but neither scenario was tested in actual use. The paired result therefore supports no superiority claim for either model and does not demonstrate that an assisted PHC service would increase uptake.

### 4.5. Digital Device Literacy and Telemedicine Perceptions

Mean digital literacy was 4.11 (SD 0.62; 95% CI 4.04–4.18). The six-item scale had alpha = 0.922, omega = 0.925, KMO = 0.899, and one-factor loadings of 0.731–0.859. Correlation results are presented in Table 5.

Digital literacy correlated negatively with home barriers (Pearson r = −0.440, 95% CI −0.527 to −0.344; Spearman rho = −0.461, both *p* < 0.001) and positively with home intention (r = 0.362, 95% CI 0.260–0.457; rho = 0.403, both *p* < 0.001).

Digital literacy was positively correlated with PHC intention (r = 0.236, 95% CI 0.126–0.340, *p* < 0.001; rho = 0.249, *p* < 0.001) but not with the PHC facilitator score (r = 0.043, 95% CI −0.071 to 0.155, *p* = 0.459; rho = 0.065, *p* = 0.264). No inference was made that the associations differed in magnitude across scenarios.

Pearson and Spearman analyses produced the same directional conclusions. Because of the selected sample, bounded scale distributions, multiple tests, and the absence of a formal comparison between dependent correlations, these associations are interpreted cautiously.

### 4.6. Adjusted Multivariable Analyses

Separate multivariable models were fitted for the matched three-item intention score for each scenario. Table 6 presents focal coefficients; complete coefficients are provided in Supplementary File S3.

Higher home barriers were independently associated with lower home intention, and higher PHC facilitator scores were associated with higher PHC intention. Digital literacy remained positively associated with both outcomes. In the full home-intention model reported in Supplementary File S3, age (B = 0.017; 95% CI 0.000 to 0.035; standardized beta = 0.095; *p* = 0.049) and residence in Gyeonggi rather than Seoul (B = 0.209; 95% CI 0.028 to 0.390; standardized beta = 0.152; *p* = 0.024) also met the prespecified *p* < 0.05 threshold. Maximum VIF was 1.64. Breusch–Pagan tests indicated heteroskedasticity for the home model (*p* < 0.001) but not the PHC model (*p* = 0.051), and Shapiro–Wilk tests indicated residual non-normality in both models (both *p* < 0.001); HC3 inference was therefore retained. These cross-sectional coefficients represent adjusted associations, not causal effects, and the small age and regional coefficients should be interpreted cautiously.

### 4.7. Model Preference and Reasons for Preferring PHC-Based Telemedicine

Of 300 participants, 128 preferred home-based telemedicine (42.7%; 95% CI 37.2–48.3%), 124 preferred PHC-based telemedicine (41.3%; 95% CI 35.9–47.0%), and 48 were unsure (16.0%; 95% CI 12.3–20.6%). These preference estimates are presented in Table 7.

Among the 124 participants preferring PHC-based telemedicine, 87 selected professional assistance (70.2%; 95% CI 61.6–77.5%), 61 communication support (49.2%; 95% CI 40.6–57.9%), 40 measurement accuracy (32.3%; 95% CI 24.7–40.9%), 36 accessibility (29.0%; 95% CI 21.8–37.6%), 16 safety (12.9%; 95% CI 8.1–19.9%), and one other reason (0.8%; 95% CI 0.1–4.4%). The corresponding multiple-response results are presented in Table 8.

These multiple-response results describe reasons selected after a stated preference; they do not show that the PHC scenario objectively improves accuracy, communication, safety, or access.

Open-ended responses also indicated concerns about independent telemedicine use, including difficulty operating devices, privacy concerns, uncertainty about accurate communication with physicians, concerns about measurement reliability, and anxiety about not receiving immediate help when problems occur. These responses are consistent with the quantitative findings that immediate assistance, measurement accuracy, and privacy were among the major perceived barriers to home-based telemedicine.

Taken together, the item-level and preference results generate hypotheses about which service features some participants may value. They do not establish that PHC-based telemedicine is superior, equivalent, more accessible, or effective in practice.

## 5. Discussion

This fixed-order, scenario-based study described different constructs for two service models rather than conducting a symmetrical evaluation of the models. Participants reported specific anticipated home-use barriers and positive anticipated PHC facilitators. These findings are best understood as item-level perceptions among a selected online sample.

For the three matched intention items, the PHC-minus-home difference was −0.030 (95% CI −0.107 to 0.047; dz = −0.045, 95% CI −0.155 to 0.071). The study therefore detected no statistically significant difference, but the analysis did not test or demonstrate equivalence. Preference estimates likewise did not show majority support for either model.

The selected sample had high education and digital literacy and very few adults aged 80 years or older. This selection likely reduced representation of the people most likely to have sensory, cognitive, access-related, or technology-related difficulties. Similar concerns about older-adult telemedicine readiness and unequal utilization have been reported elsewhere [3,4,5,13,14].

Professional assistance can be conceptualized as service scaffolding, but this mechanism was not experimentally tested. The positive PHC item ratings may reflect the content and positive framing of the scenario, fixed-order contrast effects, or genuine anticipated value of staff support. These explanations cannot be separated in the present design.

Trust and privacy concerns have been linked to continuance intentions for non-face-to-face telemedicine [15]. In this study, however, institutional trust was not measured as a separate validated construct; the PHC privacy and measurement items should, therefore, remain descriptive rather than being interpreted as evidence that public institutions confer trust.

The PHC scenario retained in-person staff contact while connecting the patient remotely to a clinician. Participants may have responded to that combination, but the survey did not measure loneliness, reassurance, actual communication quality, travel burden, waiting time, or facility accessibility. These remain implementation questions.

In adjusted analyses, higher home barriers were associated with lower home intention, while PHC facilitator ratings were associated with higher PHC intention. Digital literacy remained positively associated with both intention outcomes. Age and residence in Gyeonggi rather than Seoul were also associated with home intention in the full model, although their standardized coefficients were small and the age association was borderline (*p* = 0.049). These cross-sectional associations do not establish causation or confirm the direction proposed by the theoretical framework.

Although the bivariate digital-literacy correlations differed numerically, no formal comparison of dependent correlations was performed. The manuscript therefore does not claim that digital literacy is more important for one scenario. The restricted digital-literacy range also limits inferences about less digitally capable populations.

The findings can inform hypotheses for service design, such as testing whether measurement support, troubleshooting, and communication assistance affect uptake. They do not demonstrate that a PHC pathway will improve accessibility, reduce inequality, or produce better outcomes.

An assisted PHC pathway may merit pilot evaluation as one option alongside home-based services. Such evaluation should compare matched constructs, randomize or counterbalance scenario presentation, and measure actual use, access, satisfaction, clinical outcomes, workload, and cost.

Digital health can reproduce existing inequities when access, literacy, disability, age, and social conditions are not adequately addressed [16,17]. The present study did not directly measure inequity or changes in access; consequently, equity is discussed as a policy rationale for future research rather than an empirical outcome.

The findings should be interpreted as responses to fixed-order hypothetical descriptions, not as evidence of actual service use, sustained adoption, policy effectiveness, or clinical benefit. Real-world outcomes may also depend on travel distance, waiting time, staffing, cost, privacy conditions, clinician responsiveness, and platform performance, none of which were evaluated here.

Future research should evaluate PHC-based telemedicine in real-world implementation settings. Pilot programs could examine whether the perceived acceptability of PHC-based telemedicine translates into actual use, user satisfaction, continuity of care, clinical outcomes, and cost-effectiveness. Studies with larger and more diverse samples, including rural older adults, adults aged 80 years or older, and those with very low digital literacy, would also help determine whether the PHC-based model is generalizable across different populations and healthcare contexts.

### 5.1. Practical Implications

The item-level results identify support features that could be tested in future service-design studies, including measurement assistance, troubleshooting, and communication support. The present survey does not establish which service design should be adopted.

Pilot investigators could evaluate structured support processes in PHCs or community clinics, including pre-consultation assistance, measurement support, device guidance, connection to remote clinicians, and troubleshooting. Feasibility, staff workload, privacy, cost, and patient outcomes require prospective measurement.

Future assisted-use pilots could evaluate staff-facing interfaces, simplified workflows, hand-off functions, remote support, and confirmation of data transmission. This study did not test platform usability or determine which features improve use.

A future comparative pilot could examine whether home-based and assisted pathways serve users with different capabilities and preferences. The present selected hypothetical survey cannot determine the optimal delivery model or show that matching pathways improves acceptance or reduces exclusion.

### 5.2. Policy Implications

From a policy perspective, the results justify further evaluation of differentiated telemedicine pathways, not immediate implementation. Any assisted model would require evidence concerning staffing, clinical governance, privacy, financing, travel distance, and opportunity cost.

Future pilots could define and evaluate the role of nurses, public health personnel, or trained facilitators in device setup, measurement, troubleshooting, and communication support. Their competencies, workload, accountability, and cost would need prospective assessment.

Eligibility or referral criteria for assisted services should not be inferred from this selected sample. Future studies should include people with lower digital literacy, sensory or cognitive limitations, disability, institutional residence, rural residence, and advanced age, while also preserving home-based options for those who prefer them.

Integration with community chronic disease programs is a testable implementation hypothesis. Pilot studies should establish whether the proposed pathway is feasible and acceptable to patients, staff, and clinicians before broader policy adoption.

Any policy consideration should be preceded by implementation studies of staffing, equipment, training, workflow, privacy, financing, patient experience, clinical outcomes, provider workload, and cost-effectiveness. The positive scenario ratings alone are insufficient to justify adoption.

## 6. Conclusions

Among relatively digitally capable, community-dwelling older adults who independently completed an online survey, no statistically detectable difference was observed between matched home-based and PHC-based intention scores. This result does not establish equivalence. Home-barrier and PHC-facilitator scores were not directly comparable because their wording and constructs differed.

Participants most strongly endorsed lack of immediate help as a home-based concern and staff assistance as a PHC-based facilitator. These item-level findings generate a hypothesis that assisted delivery may be valued by some users, but they do not demonstrate actual adoption, greater accessibility, reduced inequality, or clinical benefit.

The results should not be generalized to older adults broadly, especially those unable to complete an online survey independently. Real-world, counterbalanced studies using symmetrical measures and inclusive recruitment are required before drawing implementation or policy conclusions.

Future evaluations should measure actual uptake, sustained engagement, satisfaction, clinical outcomes, privacy, staff workload, travel and waiting burden, and cost-effectiveness across diverse levels of digital capability.

## 7. Limitations and Future Research Directions

Several limitations should be considered when interpreting the findings. First, this study used a cross-sectional, scenario-based survey design. Participants evaluated hypothetical telemedicine models rather than participating in actual telemedicine services. Therefore, the findings reflect perceived barriers, perceived facilitators, acceptability, intention to use, and model preference, not actual adoption behavior, sustained use, or clinical effectiveness. Although scenario-based methods are useful for examining anticipated acceptance of emerging service models, participants’ stated willingness may differ from their behavior in real-world settings.

Second, the non-probability online sample was not representative of older adults in South Korea. Seventy-three percent had a college education or higher, mean digital literacy was 4.11/5, only 6.0% scored 3.0 or lower, and only five participants were aged 80 years or older. Generalizability is therefore limited to relatively digitally capable community-dwelling adults able to complete an online survey independently.

Third, independent online completion excluded or underrepresented older adults with very low digital literacy, limited internet access, sensory or cognitive limitations, severe disability, or a need for assistance. These groups may be particularly relevant to an assisted telemedicine model, so their exclusion could bias estimates of barriers, facilitators, intention, and preference.

Fourth, the two scenario blocks used asymmetrical framing and a fixed order: home-based care was assessed with negatively framed barriers before PHC-based care was assessed with positively framed facilitators. Scenario and item order were not randomized. Framing, contrast, acquiescence, and order effects may therefore have influenced responses, and no direct comparison between the two domain means is valid.

Fifth, the instrument was developed for this study and lacked documented expert content validation, cognitive interviewing, pilot testing, translation validation, test–retest reliability, and external construct validation. The exploratory one-factor results and internal-consistency estimates were obtained from the same sample and should not be interpreted as full psychometric validation.

Sixth, a non-significant paired test does not establish equivalence. No clinically or practically justified equivalence margin was prespecified, and no equivalence test was performed. The conclusion is limited to the absence of a statistically detectable difference in the three matched intention items.

Seventh, several CHERRIES participation and administration metrics were unavailable [18]. The authors did not receive counts of invited panel members, unique visitors, survey starters, or screening-stage exclusions; therefore, view, participation, and completion rates could not be calculated. Actual response-duration or timestamp data, incentive details, the technical method used to prevent multiple submissions, and a complete agency quality-control log, including speeding or straight-lining thresholds, were also unavailable. The final delivered dataset allowed only post-delivery checks for duplicate IDs, exact duplicate rows, completeness of core variables, and withdrawal status. Eighth, rurality and separate prior healthcare-device experience were not measured; device-use ability was embedded in the digital literacy score. Finally, the study measured hypothetical perceptions rather than actual access, use, implementation, or health outcomes. Future studies should address these limitations using inclusive recruitment, symmetrical validated measures, randomized or counterbalanced presentation, and real-world follow-up.

## Figures and Tables

**Table 1:** Participant characteristics (N = 300).

Characteristic	Value
Age, mean ± SD, years	68.93 ± 3.48 (95% CI 68.53–69.32)
Age range, years	65–85
Age group, *n* (%)	
65–69 years	188 (62.7)
70–79 years	107 (35.7)
≥80 years	5 (1.7)
Sex, *n* (%)	
Male	179 (59.7)
Female	121 (40.3)
Education, *n* (%)	
Elementary school or lower	1 (0.3)
Middle school	6 (2.0)
High school	74 (24.7)
College or higher	219 (73.0)
Living arrangement, *n* (%)	
Living alone	35 (11.7)
Living with spouse	195 (65.0)
Living with children or family	70 (23.3)
At least one chronic disease, *n* (%)	223 (74.3)
Prior awareness of telemedicine, *n* (%)	276 (92.0)
Prior use of telemedicine, *n* (%)	15 (5.0)
Digital device literacy score, mean ± SD	4.11 ± 0.62 (95% CI 4.04–4.18)
Digital device literacy score ≤ 3.0, *n* (%)	18 (6.0)
Residential region, *n* (%)	
Seoul	73 (24.3)
Gyeonggi	93 (31.0)
Metropolitan cities	56 (18.7)
Other provinces	78 (26.0)

**Table 2:** Perceived barriers to independent home-based telemedicine.

Item	Description	Mean ± SD (95% CI)
A1	Using telemedicine alone at home would be burdensome.	2.54 ± 1.00 (2.43–2.65)
A2	Using devices or smartphones would be difficult.	2.32 ± 0.98 (2.21–2.43)
A3	I would be concerned about the accuracy of measurement results.	3.07 ± 0.98 (2.96–3.18)
A4	It would be difficult to communicate my condition to the physician.	2.76 ± 0.97 (2.65–2.87)
A5	I would be concerned about privacy protection.	3.03 ± 0.99 (2.92–3.15)
A6	It would be difficult to receive immediate help if a problem occurred.	3.38 ± 1.06 (3.26–3.50)
A7	Home-based telemedicine would feel psychologically uncomfortable.	2.51 ± 0.96 (2.40–2.62)
A8	Overall, I would not want to use home-based telemedicine (descriptive only).	2.61 ± 0.99 (2.50–2.72)
Overall	Seven-item barrier composite (A1–A7)	2.80 ± 0.76 (2.71–2.89)

Note: Values are mean ± SD (95% CI). The composite includes A1–A7 only; A8 is descriptive and not included. Alpha = 0.882; omega = 0.884. Exploratory one-factor loadings for A1–A7 ranged from 0.547 to 0.811.

**Table 3:** Perceived facilitators and acceptability of PHC-based telemedicine.

Item	Description	Mean ± SD (95% CI)
B1	PHC-based telemedicine would be less burdensome.	3.29 ± 0.76 (3.21–3.38)
B2	Using devices would be easier with help from healthcare personnel.	3.95 ± 0.71 (3.87–4.03)
B3	Measurement results obtained at a PHC would be more trustworthy.	3.44 ± 0.80 (3.35–3.53)
B4	Communication with physicians would be more comfortable with PHC staff support.	3.81 ± 0.74 (3.72–3.89)
B5	PHC-based telemedicine would be trustworthy in terms of privacy protection.	3.36 ± 0.76 (3.27–3.44)
B6	It would feel safe because immediate help would be available if a problem occurred.	3.67 ± 0.81 (3.58–3.76)
B7	PHC-based telemedicine would be psychologically easier to accept.	3.56 ± 0.86 (3.47–3.66)
Overall	Exploratory seven-item facilitator composite	3.58 ± 0.58 (3.52–3.65)

Note: Values are mean ± SD (95% CI). Alpha = 0.863; omega = 0.864. Exploratory one-factor loadings ranged from 0.532 to 0.794.

**Table 4:** Paired comparison of intention to use between home-based and PHC-based telemedicine.

Analysis	Home-Based Mean ± SD (95% CI)	PHC-Based Mean ± SD (95% CI)	Mean Difference (95% CI)	t(df)	*p*	Cohen’s dz (95% CI)
Matched three-item intention score	3.911 ± 0.637 (3.839–3.984)	3.881 ± 0.658 (3.806–3.956)	−0.030 (−0.107 to 0.047)	−0.771 (299)	0.441	−0.045 (−0.155 to 0.071)

Note: Mean difference = PHC minus home. The 95% CI for Cohen’s dz was estimated using 20,000 bootstrap resamples. No equivalence margin or equivalence test was prespecified.

**Table 5:** Correlations between digital device literacy and telemedicine-related outcomes.

Outcome	Pearson r (95% CI)	*p*	Spearman Rho	*p*
Home barrier score	−0.440 (−0.527 to −0.344)	<0.001	−0.461	<0.001
PHC facilitator score	0.043 (−0.071 to 0.155)	0.459	0.065	0.264
Home intention score	0.362 (0.260 to 0.457)	<0.001	0.403	<0.001
PHC intention score	0.236 (0.126 to 0.340)	<0.001	0.249	<0.001

Note: Pearson 95% CIs used Fisher’s z transformation. Spearman correlations are sensitivity analyses. Correlation magnitudes between outcomes were not formally compared.

**Table 6:** Focal coefficients from adjusted intention-to-use models.

Outcome	Predictor	B	HC3 SE	95% CI	Standardized Beta	*p*
Home intention	Home barrier	−0.366	0.059	−0.482 to −0.251	−0.436	<0.001
Home intention	Digital literacy	0.165	0.063	0.042 to 0.288	0.161	0.009
PHC intention	PHC facilitator	0.542	0.066	0.412 to 0.671	0.475	<0.001
PHC intention	Digital literacy	0.221	0.060	0.104 to 0.338	0.208	<0.001

Note: Each model was adjusted for age, sex, education, any chronic disease, telemedicine awareness, prior telemedicine use, residential region, and living arrangement. Home model adjusted R2 = 0.293; PHC model adjusted R2 = 0.284. HC3 robust standard errors were used.

**Table 7:** Preferred telemedicine model.

Preferred Model	*n*	%	Wilson 95% CI
Home-based telemedicine	128	42.7	37.2–48.3%
PHC-based telemedicine	124	41.3	35.9–47.0%
Not sure	48	16.0	12.3–20.6%
Total	300	100.0	-

Neither option accounted for a majority of preferences, and the overlapping intervals do not support a claim that one model was preferred overall. Preferences were elicited after both scenarios had been presented in a fixed order.

**Table 8:** Reasons for preferring PHC-based telemedicine among participants who preferred the PHC-based model (*n* = 124).

Reason	*n*	%	Wilson 95% CI
Professional assistance	87	70.2	61.6–77.5%
Communication support	61	49.2	40.6–57.9%
Measurement accuracy	40	32.3	24.7–40.9%
Accessibility	36	29.0	21.8–37.6%
Safety	16	12.9	8.1–19.9%
Other	1	0.8	0.1–4.4%

Note: Multiple responses were allowed. Percentages and Wilson 95% CIs use *n* = 124 as the denominator.

## Data Availability

The data presented in this study are available on request from the corresponding author due to privacy and ethical restrictions related to survey responses from older adults. The questionnaire is provided as Supplementary File S1.

## Supplementary Materials

The following supporting information can be downloaded at https://www.mdpi.com/article/10.3390/healthcare14162660/s1. Supplementary File S1: Bilingual Survey Questionnaire Original Korean Questionnaire with English Translation Notes; Supplementary File S2: STROBE Checklist for Cross-Sectional Studies; Supplementary File S3: Full Adjusted Regression Coefficients and Diagnostics; Supplementary File S4: CHERRIES-Aligned Reporting of Available Internet-Survey Information.
