# Supplementary material for: Perceived Barriers to Home-Based Telemedicine and Facilitators of Public Health Center-Based Telemedicine Among Digitally Capable Community-Dwelling Older Adults: A Cross-Sectional Scenario-Based Survey
Source: Healthcare (Basel). 2026 Aug 21;14(16):2660. doi: 10.3390/healthcare14162660 (PMC13512222; doi:10.3390/healthcare14162660)
Supplement: Supplementary file 1 [file healthcare-14-02660-s001.zip › Supplementary_File_S1_Bilingual_Questionnaire.pdf]

Supplementary File S1. Bilingual Survey Questionnaire

Original Korean Questionnaire with English Translation Notes

Note. The original survey was administered in Korean. English translations are provided below each Korean item for review and reproducibility. Programming notes are retained where relevant.

Survey Overview / 설문 개요

Korean Field	Korean Original	English Field	English Translation
대상자	전국 만 65 세 이상 남녀, 지역사회 거주자	**Target population**	Community-dwelling men and women aged 65 years or older nationwide
샘플수	300 명	**Sample size**	300 participants
쿼터	없음	**Quota**	None

Screening Questions / 선별 문항

SQ1. 귀하의 성별은 무엇입니까? **[1 개선택]**

EN: What is your sex? [Select one]

1) 남 *(Male)*
2) 여 *(Female)*

SQ2. 귀하의 출생연도는 어떻게 되십니까? **[출생연도 입력]**

_______ 년

EN: What is your year of birth? [Enter year of birth]

_______ year

[PROG] Proceed only if the participant is aged 65 years or older.

SQ3-1. 현재 지역사회에 거주하고 있습니까? **[1 개선택]**

EN: Do you currently reside in the community? [Select one]

1) 예 *(Yes)*
2) 아니오 (조사종료) *(No (terminate survey))*

SQ3-2. 귀하의 거주 지역은 어디입니까? **[지도에서 선택]**

EN: Where is your region of residence? [Select on map]

[PROG] Display a map of the 17 provinces/metropolitan cities.

SQ3-2_recode. 거주 지역 재분류

EN: Recoded residential region

1) 서울 *(Seoul)*
2) 경기 *(Gyeonggi)*
3) 광역시 *(Metropolitan city)*
4) 기타(else) *(Other)*

Study Information and Online Consent / 연구 설명 및 온라인 동의

[PROG: 2026-0085-003_1_대상자_설명문_및_온라인동의서_Ver5.0_20260605 파일 그대로 화면제시]

EN: [PROG: Display the file 2026-0085-003_1_Participant_Information_and_Online_Consent_Form_Ver5.0_20260605 as-is.]

Korean Field	Korean Original	English Field	English Translation
연구 제목	보건소 기반 원격의료가 노년층을 위한 보완적 대안이 될 수 있을까? 가정 기반 모델과의 비교 연구	**Study title**	Can Public Health Center-Based Telemedicine Be a Complementary Alternative for Older Adults? A Comparative Study with Home-Based Models
버전	Ver. 3.0	**Version**	Ver. 3.0
작성일	2026.05.20	**Date prepared**	20 May 2026
문서 유형	대상자용 온라인 설문지	**Document type**	Online questionnaire for participants
연구책임자	장원석 교수 / 연세대학교 의과대학	**Principal investigator**	Professor Wonseuk Jang / Yonsei University College of Medicine
연구담당자	지승권	**Research staff**	Seung-Kwon Ji
연구방식	온라인 설문조사 대행업체를 통한 온라인 설문	**Research method**	Online survey administered through a professional online survey agency
모집인원	최종 유효 응답 100 명 이상 확보 목표, 최대 300 명	**Recruitment target**	Target of at least 100 valid responses, up to 300 participants

본 설문은 노인의 원격의료 이용에 대한 인식과 수용성을 조사하기 위한 학술 연구입니다. 응답 내용은 연구 목적에만 사용되며, 연구자는 성명, 연락처, 주소, 이메일 등 직접식별정보를 수집하지 않습니다. 설문은 약 30~40 분 정도 소요됩니다.

EN: This survey is an academic study designed to examine older adults' perceptions and acceptability of telemedicine. Responses will be used only for research purposes, and the researchers will not collect directly identifiable information such as name, contact information, address, or e-mail address. The survey will take approximately 30–40 minutes.

온라인 연구 참여 동의: 본인은 온라인 연구설명문을 읽고, 본 연구의 목적, 방법, 예상 소요시간, 예상되는 불편과 위험, 개인정보 보호 및 철회 권리에 대해 이해하였습니다. 본인은 본 연구에 자발적으로 참여하는 것에 동의합니다.

EN: Online research participation consent: I have read the online study information and understand the purpose, methods, expected time required, anticipated discomforts and risks, personal information protection, and right to withdraw from the study. I voluntarily agree to participate in this study.

S0. 본 연구에 자발적으로 참여하는 것에 동의하십니까? [1 개선택]

EN: Do you voluntarily agree to participate in this study? [Select one]

1) 동의함 *(Agree)*
2) 동의하지 않음 (설문 종료) *(Do not agree (terminate survey))*

S1. 본 연구의 설명문을 읽고 내용을 이해할 수 있습니까? [1 개선택]

EN: Can you read and understand the study information? [Select one]

1) 예 *(Yes)*
2) 아니오 (설문 종료) *(No (terminate survey))*

S2. 온라인 설문에 스스로 응답할 수 있습니까? [1 개선택]

EN: Can you complete the online questionnaire by yourself? [Select one]

1) 예 *(Yes)*
2) 아니오 (설문 종료) *(No (terminate survey))*

S3. 본 연구 참여에 자발적으로 동의하십니까? [1 개선택]

EN: Do you voluntarily consent to participate in this study? [Select one]

1) 예 *(Yes)*
2) 아니오 (설문 종료) *(No (terminate survey))*

S4. 설문 제출 후 철회를 요청할 때 사용할 수 있는 응답확인코드를 자발적으로 입력하시겠습니까? **[1 개선택]**

EN: Would you like to voluntarily enter a response confirmation code that may be used to request withdrawal after survey submission? [Select one]

1) 입력함: (________) *(Enter code: (________))*
2) 입력하지 않음 *(Do not enter)*

※ 응답확인코드는 연구대상자가 스스로 정하는 임의의 문자 또는 숫자 조합입니다. 이름, 전화번호, 생년월일 등 직접식별정보는 입력하지 않도록 유의해 주시기 바랍니다.

EN: ※ The response confirmation code is an arbitrary combination of letters or numbers chosen by the participant. Please do not enter directly identifiable information such as name, telephone number, or date of birth.

Demographic and Background Information / 인구사회학적 및 배경 정보

D1. 최종 학력을 선택해 주세요. **[1 개선택]**

EN: Please select your highest level of education. [Select one]

1) 초등학교 졸업 이하 *(Elementary school or lower)*
2) 중학교 졸업 *(Middle school)*
3) 고등학교 졸업 *(High school)*
4) 대학교 졸업 이상 *(College or higher)*

D2. 동거 형태를 선택해 주세요. **[1 개선택]**

EN: Please select your living arrangement. [Select one]

1) 혼자 거주 *(Living alone)*
2) 배우자와 동거 *(Living with spouse)*
3) 자녀/가족과 동거 *(Living with children or family)*
4) 기타 *(Other)*

D3. 현재 진단받아 치료 또는 관리 중인 만성질환이 있습니까? (복수 선택 가능) **[모두선택]**

EN: Do you currently have any diagnosed chronic disease that is being treated or managed? (Multiple selections allowed) [Select all that apply]

1) 고혈압 *(Hypertension)*
2) 당뇨병 *(Diabetes)*
3) 심혈관 질환 *(Cardiovascular disease)*
4) 관절·근골격계 질환 *(Joint or musculoskeletal disease)*
5) 호흡기 질환 *(Respiratory disease)*
6) 기타 ( ) *(Other ( ))*
7) 없음 *(None)*

[PROG] Option 7 can only be selected alone.

Telemedicine Awareness and Experience / 원격의료 인지 및 경험

E1. 원격의료(비대면 진료 또는 원격 모니터링)에 대해 들어본 적이 있습니까? **[1 개선택]**

EN: Have you ever heard of telemedicine (non-face-to-face medical consultation or remote monitoring)? [Select one]

1) 예 *(Yes)*
2) 아니오 *(No)*

E2. 원격의료를 실제로 이용해 본 경험이 있습니까? **[1 개선택]**

EN: Have you ever actually used telemedicine? [Select one]

1) 예 *(Yes)*
2) 아니오 *(No)*

E3. 이용해 본 원격의료 유형은 무엇입니까? (복수 선택 가능) **[모두선택]**

EN: What type of telemedicine have you used? (Multiple selections allowed) [Select all that apply]

1) 전화 또는 영상 진료 *(Telephone or video consultation)*
2) 스마트폰/앱 기반 진료 *(Smartphone/app-based consultation)*
3) 혈압·혈당 등 건강정보 원격 전송 *(Remote transmission of health information such as blood pressure or blood glucose)*
4) 기타 ( ) *(Other ( ))*
[PROG] Show only to respondents who answered E2 = 1 (Yes).

[PROG: 10 초 제어]
EN: [PROG: 10-second control.]

Scenario Descriptions / 시나리오 설명

아래는 두 가지 원격의료 방식에 대한 설명입니다. 각 설명을 충분히 읽고 이해하신 후, 이후 문항에 응답해 주시기 바랍니다.
EN: The following descriptions present two types of telemedicine. Please read and understand each description carefully before answering the following questions.

[시나리오 A] 재택 기반 원격의료
노인이 가정에서 혼자 스마트폰, 컴퓨터 또는 의료기기를 사용하여 혈압·혈당 등을 측정하고, 그 결과를 전송하거나 영상 또는 전화로 의사와 진료하는 방식입니다. 이 과정에서 의료전문인력의 직접적인 도움은 제공되지 않습니다.
EN: [Scenario A] Home-Based Telemedicine
An older adult independently uses a smartphone, computer, or medical device at home to measure blood pressure, blood glucose, or other health information, transmits the results, and consults with a physician by video or telephone. Direct assistance from healthcare personnel is not provided during this process.

[시나리오 B] 보건소 기반 원격의료
노인이 가까운 보건소에 방문하여 간호사 등 의료전문인력의 도움을 받아 건강 정보를 측정하고, 보건소에서 원격으로 의사와 연결되어 진료를 받는 방식입니다.
EN: [Scenario B] Public Health Center-Based Telemedicine
An older adult visits a nearby public health center, measures health information with assistance from healthcare personnel such as nurses, and receives care by being remotely connected to a physician at the public health center.

A1-1. 다음 문항은 원격의료 이용 시 느낄 수 있는 부담감이나 거부감에 관한 질문입니다. 각 문항에 대해 가장 적절하다고 생각되는 번호를 선택해 주십시오. [행별 1 개선택]
EN: A1-1. The following questions ask about the burden or resistance that may be felt when using telemedicine. Please select the number that best applies to each item. [Select one per row]

Item	Korean Original	English Translation	Response Scale
**A1**	집에서 혼자 원격의료를 이용하는 것은 부담스럽다.	Using telemedicine alone at home would be burdensome.	1–5 Likert scale<br>1 = Strongly disagree<br>2 = Disagree<br>3 = Neutral<br>4 = Agree<br>5 = Strongly agree
**A2**	기기나 스마트폰 조작이 어려울 것 같다.	Using devices or smartphones would be difficult.	1 2 3 4 5
**A3**	측정 결과의 정확성이 걱정된다.	I would be concerned about the accuracy of measurement results.	1 2 3 4 5
**A4**	원격진료 중 의사에게 내 상태를 잘 전달하기 어렵다.	It would be difficult to communicate my condition to the physician during telemedicine.	1 2 3 4 5
**A5**	개인정보 보호가 걱정된다.	I would be concerned about privacy protection.	1 2 3 4 5
**A6**	문제가 발생했을 때 즉시 도움을 받기 어렵다.	It would be difficult to receive immediate help if a problem occurred.	1 2 3 4 5
**A7**	재택 원격의료는 심리적으로 거부감이 든다.	Home-based telemedicine would feel psychologically uncomfortable.	1 2 3 4 5

Item	Korean Original	English Translation	Response Scale
**A8**	전반적으로 재택 원격의료를 이용하고 싶지 않다.	Overall, I would not want to use home-based telemedicine.	1 2 3 4 5

B1-1. 다음 문항은 원격의료 이용 시 느낄 수 있는 부담감이나 거부감에 관한 질문입니다. 각 문항에 대해 가장 적절하다고 생각되는 번호를 선택해 주십시오. [행별 1 개선택]

EN: B1-1. The following questions ask about the burden or resistance that may be felt when using telemedicine. Please select the number that best applies to each item. [Select one per row]

Item	Korean Original	English Translation	Response Scale
**B1**	보건소에서 진행하는 원격의료는 부담이 덜하다.	PHC-based telemedicine would be less burdensome.	1–5 Likert scale<br>1 = Strongly disagree<br>2 = Disagree<br>3 = Neutral<br>4 = Agree<br>5 = Strongly agree
**B2**	의료전문인력이 도와주면 기기 사용이 쉬울 것 같다.	Using devices would be easier with help from healthcare personnel.	1 2 3 4 5
**B3**	보건소에서 측정한 결과는 더 신뢰할 수 있을 것 같다.	Measurement results obtained at a public health center would be more trustworthy.	1 2 3 4 5
**B4**	보건소 인력이 의사소통을 도와주면 진료가 더 편할 것 같다.	Communication with physicians would be more comfortable if public health center staff assist with communication.	1 2 3 4 5
**B5**	보건소 기반 원격의료는 개인정보 보호 측면에서 신뢰된다.	PHC-based telemedicine would be trustworthy in terms of privacy protection.	1 2 3 4 5
**B6**	문제가 생겼을 때 즉시 도움을 받을 수 있어 안전하다고 느낀다.	It would feel safe because immediate help would be available if a problem occurred.	1 2 3 4 5
**B7**	보건소 기반 원격의료는 심리적으로 수용하기 쉽다.	PHC-based telemedicine would be psychologically easier to accept.	1 2 3 4 5

A2-1. 다음 문항은 향후 원격의료를 이용할 의향에 대한 질문입니다. [행별 1 개선택]

EN: A2-1. The following questions ask about your intention to use telemedicine in the future. [Select one per row]

Item	Korean Original	English Translation	Response Scale
**A9**	필요하다면 재택 원격의료를 이용할 의향이 있다.	I would be willing to use home-based telemedicine if needed.	1–5 Likert scale<br>1 = Strongly disagree<br>2 = Disagree<br>3 = Neutral<br>4 = Agree<br>5 = Strongly agree
**A10**	주변에서 추천한다면 재택 원격의료를 시도해 볼 수 있다.	I could try home-based telemedicine if people around me recommended it.	1 2 3 4 5
**A11**	비용이 적절하다면 재택 원격의료를 이용할 수 있다.	I could use home-based telemedicine if the cost was appropriate.	1 2 3 4 5
**A12**	향후 만성질환 관리에 재택 원격의료가 도움이 될 것 같다.	Home-based telemedicine would be helpful for managing chronic diseases in the future.	1 2 3 4 5

B2-1. 다음 문항은 향후 원격의료를 이용할 의향에 대한 질문입니다. [행별 1 개선택]

EN: B2-1. The following questions ask about your intention to use telemedicine in the future. [Select one per row]

Item	Korean Original	English Translation	Response Scale

Item	Korean Original	English Translation	Response Scale
**B8**	필요하다면 보건소 기반 원격의료를 이용할 의향이 있다.	I would be willing to use PHC-based telemedicine if needed.	1–5 Likert scale<br>1 = Strongly disagree<br>2 = Disagree<br>3 = Neutral<br>4 = Agree<br>5 = Strongly agree
**B9**	보건소가 가까우면 정기적으로 이용할 수 있다.	I could use it regularly if the public health center was nearby.	1 2 3 4 5
**B10**	비용이 적절하다면 보건소 기반 원격의료를 이용할 수 있다.	I could use PHC-based telemedicine if the cost was appropriate.	1 2 3 4 5
**B11**	향후 만성질환 관리에 보건소 기반 원격의료가 도움이 될 것 같다.	PHC-based telemedicine would be helpful for managing chronic diseases in the future.	1 2 3 4 5

C. 다음 문항은 디지털 기기 활용 능력에 대한 질문입니다. [행별 1 개선택]

EN: C. The following questions ask about digital device literacy. [Select one per row]

Item	Korean Original	English Translation	Response Scale
**C1**	스마트폰 사용이 익숙하다.	I am familiar with using a smartphone.	1–5 Likert scale<br>1 = Strongly disagree<br>2 = Disagree<br>3 = Neutral<br>4 = Agree<br>5 = Strongly agree
**C2**	모바일 앱을 스스로 설치하고 사용할 수 있다.	I can install and use mobile applications by myself.	1 2 3 4 5
**C3**	문자메시지나 메신저 사용에 불편함이 없다.	I have no difficulty using text messages or mobile messengers.	1 2 3 4 5
**C4**	혈압계·혈당계 등 측정기기를 스스로 사용할 수 있다.	I can use measurement devices such as a blood pressure monitor or blood glucose meter by myself.	1 2 3 4 5
**C5**	인터넷 접속 및 검색을 혼자 할 수 있다.	I can access and search the internet by myself.	1 2 3 4 5
**C6**	전반적으로 디지털 기기 사용에 자신이 있다.	Overall, I am confident in using digital devices.	1 2 3 4 5

Model Preference, Open-Ended Response, and Withdrawal / 선호도, 자유응답 및 철회

P1. 재택 기반 원격의료와 보건소 기반 원격의료 중, 더 선호하는 방식은 무엇입니까? [1 개선택]

EN: Which model do you prefer between home-based telemedicine and PHC-based telemedicine? [Select one]

1) 재택 기반 원격의료 *(Home-based telemedicine)*
2) 보건소 기반 원격의료 *(PHC-based telemedicine)*
3) 잘 모르겠다 *(Not sure)*

P2. 보건소 기반 원격의료를 선호한다면, 그 가장 큰 이유는 무엇입니까? (최대 2 개 선택) [순위형으로 최대 2 순위까지 선택]

EN: If you prefer PHC-based telemedicine, what is the main reason? (Select up to two) [Rank up to second priority]

1) 접근성이 좋다 *(Better accessibility)*
2) 의료전문인력의 도움을 받을 수 있다 *(Ability to receive help from healthcare personnel)*
3) 측정 결과의 정확성이 높을 것 같다 *(Measurement results would be more accurate)*

4) 의사소통이 원활할 것 같다 *(Communication would be smoother)*
5) 안전할 것 같다 *(It would feel safer)*
6) 기타 ( ) *(Other ( ))*

P3. **원격의료를 이용할 때 가장 우려되는 점이 있다면 자유롭게 작성해 주십시오. (자유기술) [서술형]**

EN: If you have any major concerns when using telemedicine, please describe them freely. (Open-ended) [Descriptive response]

F. **지금까지 응답하신 설문 데이터를 철회하고자 하시는 경우, 앞서 입력하신 응답확인코드를 입력해 주세요. [1 개선택]**

EN: If you wish to withdraw the survey data you have submitted so far, please enter the response confirmation code you entered earlier. [Select one]

1) 철회 요청(응답확인 코드: _________) *(Request withdrawal (response confirmation code: _________))*
2) 데이터 사용에 동의합니다. *(I agree to the use of my data.)*
