# Supplementary material for: Perceived Barriers to Home-Based Telemedicine and Facilitators of Public Health Center-Based Telemedicine Among Digitally Capable Community-Dwelling Older Adults: A Cross-Sectional Scenario-Based Survey
Source: Healthcare (Basel). 2026 Aug 21;14(16):2660. doi: 10.3390/healthcare14162660 (PMC13512222; doi:10.3390/healthcare14162660)
Supplement: Supplementary file 1 [file healthcare-14-02660-s001.zip › Supplementary_File_S2_STROBE_Checklist.pdf]

Supplementary File S2. STROBE Checklist for Cross-Sectional Studies

Manuscript ID: healthcare-4463725

Item	Recommendation	Reported in revised manuscript
1(a)	Indicate the study design in the title or abstract	Title; structured Abstract
1(b)	Provide an informative and balanced abstract	Structured Abstract
2	Explain scientific background and rationale	Section 1
3	State specific objectives and hypotheses	End of Section 1; Sections 2.1-2.4
4	Present key elements of study design early	Abstract; Section 3.1
5	Describe setting, locations, and dates	Sections 3.1-3.2: South Korea; online panel; 1-8 July 2026
6(a)	Give eligibility criteria and participant selection	Section 3.2; Figure 2
7	Define outcomes, predictors, confounders, and effect modifiers	Sections 3.3-3.5
8	Give data sources and measurement details	Sections 3.3-3.4; Supplementary File S1
9	Describe efforts to address potential bias	Sections 3.1-3.2 and 3.5; Section 7
10	Explain how study size was arrived at	Section 3.2: protocol range and achieved-sample sensitivity
11	Explain handling of quantitative variables	Sections 3.4-3.5
12(a)	Describe statistical methods and confounding control	Section 3.5; Table 6; Supplementary File S3
12(b)	Describe subgroup and interaction analyses	Not performed; reasons restricted to PHC-preferring participants
12(c)	Explain how missing data were addressed	Section 3.5: complete core variables; no imputation
12(d)	Describe methods accounting for sampling strategy	Non-probability online panel; no survey weights available
12(e)	Describe sensitivity analyses	Section 3.5: Spearman correlations and bootstrap dz interval
13(a)	Report numbers at each study stage	Figure 2; Section 3.2; unavailable upstream counts identified
13(b)	Give reasons for non-participation	Not available from agency; stated in Section 3.2 and Figure 2
13(c)	Consider a flow diagram	Figure 2
14(a)	Describe participant characteristics and confounders	Table 1; Section 4.1
14(b)	Indicate missing data for each variable	Section 3.2 and Figure 2: zero missing core responses
15	Report outcome data	Sections 4.2-4.7; Tables 2-8
16(a)	Give unadjusted and adjusted estimates with precision	Tables 4-6; Supplementary File S3
16(b)	Report category boundaries	Sections 3.4-3.5; Table 1
16(c)	Translate relative risk estimates when relevant	Not applicable; continuous outcomes
17	Report other analyses	Sections 4.3-4.6; Tables 4-6
18	Summarize key results	Opening of Section 5; Section 6
19	Discuss limitations and likely bias	Section 7
20	Give a cautious overall interpretation	Sections 5-6
21	Discuss generalizability	Sections 4.1, 5, 6, and 7
22	State funding and funder role	Funding statement

Items not applicable or unavailable are identified rather than omitted.
