# Supplementary material for: Perceived Barriers to Home-Based Telemedicine and Facilitators of Public Health Center-Based Telemedicine Among Digitally Capable Community-Dwelling Older Adults: A Cross-Sectional Scenario-Based Survey
Source: Healthcare (Basel). 2026 Aug 21;14(16):2660. doi: 10.3390/healthcare14162660 (PMC13512222; doi:10.3390/healthcare14162660)
Supplement: Supplementary file 1 [file healthcare-14-02660-s001.zip › Supplementary_File_S3_Adjusted_Regression.pdf]

Supplementary File S3. Full Adjusted Regression Coefficients and Diagnostics

Manuscript ID: healthcare-4463725

Two ordinary least-squares models were fitted for matched three-item intention scores. HC3 heteroskedasticity-robust standard errors, two-sided p values, 95% confidence intervals, standardized coefficients, and variance inflation factors (VIFs) are reported. Reference categories were male, less than college education, no chronic disease, no awareness, no prior use, Seoul, and the reference living-arrangement category.

Home-based intention-to-use model

Predictor	B	HC3 SE	95% CI	Std. beta	t	p	VIF
Intercept	2.880	0.666	1.570 to 4.190	-	4.327	<0.001	-
Home barrier score	-0.366	0.059	-0.482 to -0.251	-0.436	-6.229	<0.001	1.302
Digital literacy	0.165	0.063	0.042 to 0.288	0.161	2.632	0.009	1.328
Age (years)	0.017	0.009	0.000 to 0.035	0.095	1.981	0.049	1.059
Female	0.105	0.069	-0.030 to 0.241	0.081	1.526	0.128	1.250
College or higher	0.120	0.080	-0.037 to 0.277	0.084	1.507	0.133	1.206
Any chronic disease	0.032	0.079	-0.124 to 0.187	0.022	0.399	0.690	1.133
Telemedicine awareness	-0.044	0.186	-0.411 to 0.323	-0.019	-0.236	0.814	1.115
Prior telemedicine use	0.011	0.145	-0.274 to 0.296	0.004	0.078	0.938	1.038
Region: Gyeonggi	0.209	0.092	0.028 to 0.390	0.152	2.277	0.024	1.617
Region: metropolitan city	-0.004	0.105	-0.210 to 0.202	-0.002	-0.037	0.970	1.500
Region: other province	0.141	0.103	-0.062 to 0.345	0.097	1.367	0.173	1.639
Living alone	-0.100	0.108	-0.312 to 0.112	-0.051	-0.932	0.352	1.149
Living with family	-0.109	0.085	-0.277 to 0.059	-0.073	-1.279	0.202	1.109

Diagnostic	Value
N	300
Predictors	13
R-squared	0.323
Adjusted R-squared	0.293
Model F	10.513
Model p	<0.001
Maximum VIF	1.639
Breusch-Pagan chi-square	36.146
Breusch-Pagan p	<0.001
Residual Shapiro-Wilk W	0.979
Residual Shapiro-Wilk p	<0.001

PHC-based intention-to-use model

Predictor	B	HC3 SE	95% CI	Std. beta	t	p	VIF
Intercept	0.319	0.714	-1.087 to 1.725	-	0.446	0.656	-
PHC facilitator score	0.542	0.066	0.412 to 0.671	0.475	8.243	<0.001	1.024
Digital literacy	0.221	0.060	0.104 to 0.338	0.208	3.713	<0.001	1.114
Age (years)	0.010	0.009	-0.007 to 0.027	0.052	1.125	0.261	1.056

Predictor	B	HC3 SE	95% CI	Std. beta	t	p	VIF
Female	0.050	0.069	-0.086 to 0.186	0.037	0.727	0.468	1.248
College or higher	-0.085	0.079	-0.241 to 0.071	-0.057	-1.069	0.286	1.207
Any chronic disease	0.109	0.077	-0.043 to 0.261	0.073	1.413	0.159	1.110
Telemedicine awareness	0.048	0.152	-0.251 to 0.347	0.020	0.314	0.754	1.097
Prior telemedicine use	0.089	0.111	-0.131 to 0.308	0.029	0.797	0.426	1.040
Region: Gyeonggi	0.011	0.090	-0.167 to 0.189	0.008	0.126	0.900	1.617
Region: metropolitan city	-0.165	0.098	-0.358 to 0.028	-0.098	-1.686	0.093	1.501
Region: other province	-0.017	0.094	-0.201 to 0.167	-0.011	-0.184	0.854	1.637
Living alone	-0.081	0.116	-0.309 to 0.147	-0.040	-0.699	0.485	1.153
Living with family	-0.055	0.080	-0.211 to 0.102	-0.035	-0.690	0.491	1.120

Diagnostic	Value
N	300
Predictors	13
R-squared	0.315
Adjusted R-squared	0.284
Model F	10.133
Model p	<0.001
Maximum VIF	1.637
Breusch-Pagan chi-square	22.267
Breusch-Pagan p	0.051
Residual Shapiro-Wilk W	0.982
Residual Shapiro-Wilk p	<0.001

Interpretation note: residual normality tests were significant in both models and the home model showed heteroskedasticity; HC3 robust inference was retained. Coefficients are cross-sectional adjusted associations, not causal effects. Rurality and a separate prior healthcare-device-experience variable were not collected.
