# Supplementary material for: Perceived Barriers to Home-Based Telemedicine and Facilitators of Public Health Center-Based Telemedicine Among Digitally Capable Community-Dwelling Older Adults: A Cross-Sectional Scenario-Based Survey
Source: Healthcare (Basel). 2026 Aug 21;14(16):2660. doi: 10.3390/healthcare14162660 (PMC13512222; doi:10.3390/healthcare14162660)
Supplement: Supplementary file 1 [file healthcare-14-02660-s001.zip › Supplementary_File_S4_CHERRIES_Available_Information.pdf]

SUPPLEMENTARY FILE S4

CHERRIES-Aligned Reporting of Available Internet-Survey Information

Evidence boundary: existing survey materials and final delivered dataset only

Manuscript ID: healthcare-4463725
Study: Cross-sectional, fixed-order, scenario-based online survey of 300 community-dwelling adults aged 65 years or older
Purpose: Document what can and cannot be reported without reconstructing missing survey-platform paradata

Scope and interpretation

This supplement maps the information available in the agency survey-programming document, the agency-delivered final deidentified dataset, and the current manuscript to CHERRIES reporting domains. It does not infer invitation, visitor, start, exclusion, or timing counts from sequential case identifiers. Items not documented in the available survey materials are marked as not available or not calculable.

Category	Reporting item	Information supported by available materials	Status
**Design**	Target population and sampling frame	Community-dwelling adults aged 65 years or older in South Korea; online survey administered by a professional survey agency. The sample was non-probability and no quota was used.	Available
**Design**	Survey design	Cross-sectional, scenario-based, within-subject online survey. Each participant evaluated home-based and PHC-based scenarios.	Available
**Ethics**	Ethics approval	Not reassessed in this survey-material-based supplement. The journal's required ethics statement remains in the main manuscript.	Main text
**Ethics**	Electronic consent	The programmed questionnaire presented study information and required consent, understanding of the information, ability to self-complete, and voluntary participation before the main survey.	Available
**Ethics**	Data protection	The survey introduction stated that direct identifiers were not requested. The research dataset delivered to the authors was deidentified. The optional response-confirmation code and free-text field should still be handled as potentially identifying.	Partial
**Development**	Questionnaire development and pretesting	The study-specific questionnaire and its theoretical basis are described in the manuscript. No documentation was available for expert content-validity rating, cognitive interviews, pilot testing, translation validation, test-retest assessment, or technical usability testing.	Partial

Category	Reporting item	Information supported by available materials	Status
**Recruitment**	Open or closed survey	The available materials state that a professional survey agency administered the survey but do not document whether access was technically open, closed, password-protected, or account-restricted.	Not available
**Recruitment**	Initial contact mode	The invitation channel and first-contact method were not included in the survey materials delivered to the authors.	Not available
**Recruitment**	Advertising or invitation wording	No invitation text, advertisement, mailing-list description, or placement record was provided.	Not available
**Administration**	Web or email administration	The survey was administered online through a survey agency. The available materials do not document the platform name or the technical response-capture method.	Partial
**Administration**	Survey context	A study-specific questionnaire for research on perceived barriers, facilitators, intention, digital literacy, preference, and concerns regarding two hypothetical telemedicine models.	Available
**Administration**	Stated expected duration	The survey introduction stated an expected completion time of approximately 30-40 minutes. Actual response-duration data were not included in the delivered dataset.	Available
**Administration**	Voluntary or mandatory participation	Participation was voluntary at the consent stage. Whether every subsequent response was enforced by client-side or server-side validation was not documented.	Partial
**Administration**	Incentives	Incentive type, value, and payment conditions were not stated in the survey-programming document or final dataset available for this supplement.	Not available
**Administration**	Field period	1 July to 8 July 2026, as reported in the manuscript.	Available
**Administration**	Randomization	No randomization or counterbalancing was used. Home-based content preceded PHC-based content in a fixed order.	Available
**Administration**	Adaptive questioning	Programmed branching terminated ineligible or nonconsenting respondents. The prior-telemedicine-type question was shown only to respondents reporting prior use. Other detailed routing logs were not provided.	Partial
**Administration**	Items and screens	The full questionnaire is provided as Supplementary File S1. The exact number of web pages/screens and the distribution of items across screens were not documented in the delivered materials.	Partial
**Administration**	Reading/display control	The programming document records a 10-second control immediately before the first scenario-related response block. Further implementation details were not provided.	Partial
**Administration**	Completeness checking	All 300 delivered records were complete for the core analytic variables. The technical mechanism used to require or validate responses during administration was not documented.	Partial
**Administration**	Review or change of answers	The availability of a back button or pre-submission review function was not documented.	Not available
**Participation**	Invited persons and unique visitors	Counts of invited panel members and unique survey visitors were not provided.	Not available

Category	Reporting item	Information supported by available materials	Status
**Participation**	Survey openings and starts	Counts of individuals opening the survey, reaching consent, or starting the main questionnaire were not provided.	Not available
**Participation**	Screening-stage exclusions	The survey program contained termination rules, but the number excluded at each screening step was not provided.	Not available
**Participation**	View rate	Not calculable because the required visitor and view counts were unavailable.	Not calculable
**Participation**	Participation rate	Not calculable because the denominator of unique eligible or consenting visitors was unavailable.	Not calculable
**Participation**	Completion rate	Not calculable because the number of persons who started the survey was unavailable. The 300 records represent the final valid dataset, not a platform-level completion denominator.	Not calculable
**Multiple entries**	Technical prevention	Cookies, IP checks, account authentication, device fingerprinting, or other platform-level duplicate-prevention methods were not documented.	Not available
**Multiple entries**	Post-delivery duplicate checks	The final dataset contained 300 unique case IDs and no exact duplicate full rows. These checks do not establish which technical controls were used during fielding.	Available
**Analysis**	Incomplete questionnaires	Only the final valid dataset was delivered. Core analytic variables were complete in all 300 records; incomplete or terminated questionnaires were not supplied.	Partial
**Analysis**	Response-time checks	Actual response-duration and timestamp data were absent, so speeding thresholds or atypical completion times could not be assessed by the authors.	Not available
**Analysis**	Straight-lining and agency quality control	The complete agency quality-control log and prespecified speeding, straight-lining, or inconsistency thresholds were not provided.	Not available
**Analysis**	Author exclusions and imputation	All 300 delivered valid records were analyzed. The authors excluded no additional records, and no imputation was performed because core analytic variables were complete.	Available
**Analysis**	Withdrawal status	The final withdrawal/data-use item showed 300 data-use consents and no withdrawal requests.	Available

Status key: Available = directly supported; Partial = some information is supported but technical or process details are missing; Not available = not present in delivered materials; Not calculable = required denominator or numerator is absent; Main text = retained in the manuscript rather than reassessed here.

Verified final-dataset counts

Verified item	Result
Final valid records delivered	**300**
Programmed eligibility/consent/self-completion conditions in final dataset	**All 300 passed**
Core analytic-variable missing values	**0**

Verified item	Result
Duplicate case IDs	**0**
Exact duplicate full rows	**0**
Withdrawal requests in final data-use item	**0**
Additional records excluded by the authors after delivery	**0**
Invited, unique visitors, starts, and screening exclusions	**Not provided**
CHERRIES view, participation, and completion rates	**Not calculable**

Recommended manuscript reporting boundary

Report directly: the programmed eligibility/consent gates, no-quota design, fixed order, documented 10-second control, final N = 300, final-dataset completeness, duplicate checks, and withdrawal status.

Do not estimate: invitation, visitor, start, screening-exclusion, response-time, incentive, or platform quality-control metrics. Do not use case-ID gaps to reconstruct participant flow.

Materials used

Survey programming — Agency survey-programming document for the digital telemedicine questionnaire, dated 20 May 2026

Final dataset — Agency-delivered Excel workbook; Raw sheet; 300 records

Current manuscript — manuscript.v5.docx(2).docx; used for the reported field period and current methodological descriptions

Reporting framework — Eysenbach G. Improving the Quality of Web Surveys: The Checklist for Reporting Results of Internet E-Surveys (CHERRIES). J Med Internet Res. 2004;6(3):e34. doi:10.2196/jmir.6.3.e34
